# Inline Hot Rolling of Al-5%Mg Strip Cast Using an Unequal Diameter Twin-Roll Caster

**DOI:** 10.3390/ma17143598

**Published:** 2024-07-21

**Authors:** Toshio Haga, Masataka Furukawa

**Affiliations:** 1 Department of Mechanical Engineering, Faculty of Engineering, Osaka Institute of Technology, 5-16-1, Omiya, Asahi-ku, Osaka 535-8585, Japan; 2 Graduate School, Osaka Institute of Technology, 5-16-1, Omiya, Asahi-ku, Osaka 535-8585, Japan; m1m22437@oit.ac.jp

**Keywords:** unequal diameter twin-roll caster, inline hot rolling, Al-Mg alloy, surface crack, anisotropy of elongation

## Abstract

One advantage of twin-roll casting for aluminum alloys is that hot rolling can be omitted, thus shortening the process. The effect of inline hot rolling on the anisotropy of the mechanical properties, especially the elongation, of the roll-cast strip has not been investigated. In a high-speed twin-roll caster, inline hot rolling forms the metal shape before the temperature of the cast strip decreases below the temperature needed for hot rolling. In this study, inline hot rolling of Al-5%Mg strips cast using an unequal diameter twin-roll caster was performed to validate the technique and evaluate its ability to reduce surface cracking and improve the elongation anisotropy. A rolling speed of 30 m/min was used, and the effects of temperature and thickness reduction during inline hot rolling on the surface and mechanical properties were investigated. Inline hot rolling was found to effectively reduce the formation of surface cracks and the anisotropy of the mechanical properties.

## 1. Introduction

An advantage of a conventional twin-roll caster for aluminum alloys (CTRCA) over direct chill (DC) casting and the hot rolling process is a shortening of the process [1,2]. Strips cast using a CTRCA are coiled, and the coiled strips are cold-rolled [3,4,5,6,7,8,9,10,11,12,13]. Hot rolling is not usually conducted on strips cast using a CTRCA. Omitting hot rolling is thought to be an advantage of a CTRCA as it reduces both the processing time and cost. 

It is reported that roll-cast aluminum alloy strips have anisotropic mechanical properties between the casting and lateral directions [14,15,16,17,18,19,20,21,22]. Heat treatment conditions [15,16], heat treatment and cold rolling [17,18,19,20], reduced cold rolling [21], and differential speed rolling [22,23] have been tested as means of reducing the anisotropy of the mechanical properties, although these methods lead to an increase in process complexity. It is reported that the mechanical properties of a roll-cast strip are inferior to those of a strip made by DC casting and the hot rolling process, and the mechanical properties of a roll-cast strip can be improved by hot rolling [24,25]. However, the effect of hot rolling on the anisotropy of the mechanical properties of a roll-cast strip has not yet been reported.

Since hot rolling is not used as a shortcut in twin-roll casting, its effect on the roll-cast strip, for example, the surface condition and mechanical properties, has not been clarified. Hot rolling is classified into offline hot rolling (OHR) and inline hot rolling (IHR) [26]. OHR is the conventional popular hot rolling method. When OHR is conducted, a mill for hot rolling and a heater are needed. In IHR, the as-cast strip is immediately hot-rolled, and equipment for controlling the temperature may be needed instead of the heater. In either method, the mill for hot rolling is essential. IHR is conducted as part of a processing sequence, and this may be an advantage of IHR over OHR and other processes. 

The disadvantage of a CTRCA is low productivity due to the low casting speed. The casting speed of a CTRCA is less than 2 m/min and about 1 m/min is typical. A custom-made unequal-diameter twin-roll caster (UDTRC) [27,28], which is a high-speed twin-roll caster for aluminum alloys (HSTRCA) [29,30,31,32], can cast a strip at speeds ranging from 10 to 60 m/min, thus increasing productivity. The productivity of the UDTRC is 30 times greater than that of a CTRCA when the roll speed of the CTRC and the UDTRC are 1 m/min and 30 m/min, respectively. It is considered that it is more economical to cast strips using a UDTRTC than using a CTRCA. 

In IHR, the distance between the caster and the mill for hot rolling is about 20 m, considering the conveying equipment and the temperature control unit. In a CTRCA, when the casting speed is 1 m/min, it takes 20 min to convey the strip from the CTRCA to the hot rolling mill. There is concern that this delay may decrease the strip temperature below the temperature appropriate for hot rolling. In IHR using a UDTRC, the strip travels from the caster to the hot rolling mill in 40 s when the roll speed is 30 m/min. In this scenario, the temperature of the strip may be sufficiently high for hot rolling.

In the present study, IHR using a UDTRC was performed to examine its effectiveness in reducing surface cracking and elongation anisotropy between the casting and lateral directions. First, the strip temperature drop during conveying from the caster to the hot rolling mill was investigated. For comparison, strips were also produced by both cold rolling and OHR followed by cold rolling. 

## 2. Experimental Conditions

**Figure 1:**
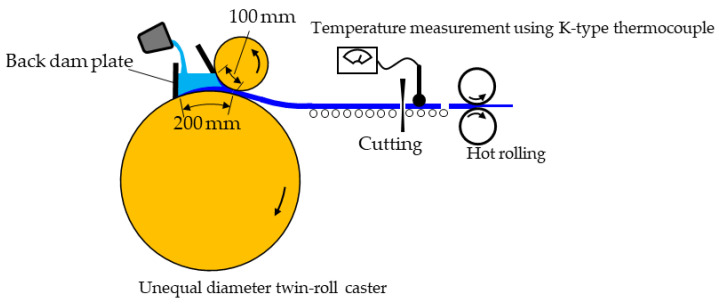
Schematic diagram of experimental equipment for inline hot rolling.

A schematic diagram of the experimental equipment for IHR is shown in Figure 1. A time–temperature curve for the cast strip was measured to determine if IHR could be conducted in the hot-working zone. Cast strips with a width of 100 mm, a thickness of 3.7 mm, and a length of 8 m were cut to a length of 300 mm, their temperature was measured, and then they were cooled in air until they reached the designated temperature for hot rolling. The cut strips were hot-rolled at the designated temperature and their thickness was reduced. In the present study, the IHR temperature was 350 or 450 °C. The mill was small, and the motor power was insufficient to roll the full width of the strip. Thus, only the 40 mm wide center region of the strip was rolled. The thickness reduction during hot rolling was 19, 27, or 36%. The hot-rolled strips were cooled to room temperature in air and then cold rolled down to 0.5 or 1.0 mm. The rolling direction was the same as the casting direction. Penetrant tests were conducted on the cast strips, the IHR strips, and the cold-rolled strips to investigate surface cracks. The cold-rolled strips were annealed at 360 °C for 90 min. Test pieces for tensile testing were cut from the annealed strips using a wire cutter. Tensile testing was performed in both the casting and lateral directions using the universal testing machine (AG-I/100 kN, SIMADZU, Nakagyo-ku, Kyoto, Japan). The tensile test pieces had a gage length of 8 mm, a gage width of 4 mm, and full length of 40 mm. The cross-head speed during the tensile tests was 1 mm/min. Seven test pieces were tested, those that gave the maximum and minimum elongations were excluded, and the mechanical properties were determined from the remaining five. The penetrant and tensile tests were conducted on the cold-rolled strips and the OHR + the cold-rolled strips for comparison with the IHR strips. The cast strips were heated to the designated temperature and kept for 0.5 h in an electric furnace, after which OHR was conducted. The microstructure of the strips was observed using optical microscopy. 

A custom-made UDTRC was used in the present study. Its upper and lower rolls were made of copper with diameters of 300 and 1000 mm, respectively; each was 100 mm wide. Parting material was not used on the roll surface because the cast strips did not stick to the roll surface. The roll speed and load were 30 m/min and 13 N/mm, respectively. The initial roll gap was 1 mm, and it increased depending on strip thickness. The solidification lengths (contact lengths between the aluminum alloy and roll) for the upper and lower rolls were 100 and 200 mm, respectively. After the rolls in the UDTRC reached the designated rotation speed, molten metal was poured at 720 °C. A small mill equipped with 70 mm diameter rolls (DBR70, Daito Seisakuzyo, Katsushika-ku, Tokyo, Japan) was used for hot rolling. The chemical composition of the Al-5%Mg alloy used in the present study is shown in Table 1.

## 3. Results and Discussion

### 3.1. Time and Cast Strip Temperature

**Figure 2:**
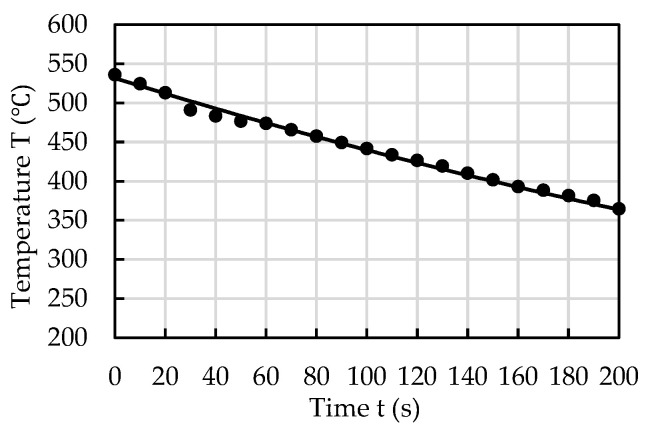
Time–temperature cooling curve for the cast strip. The roll speed was 30 m/min.

Considering the size of the conveyer and the temperature-controlling equipment, the distance between the caster and mill was assumed to be 20 m. At a roll speed of 30 m/min, the cast strip would take 40 s to move this 20 m distance, and the thickness of the cast strips was 3.7 mm. A time–temperature cooling curve was measured to determine the possibility of IHR at the required temperature. The hot rolling temperature for aluminum alloys is usually higher than 350 °C. The time–temperature cooling curve for the cast strip is shown in Figure 2. The strip temperature after moving 20 m, which took 40 s, was found to be 483 °C. This is high enough for IHR after moving from the caster to the mill, which indicates that IHR is commercially viable. 

### 3.2. Reduction in Surface Cracking on Strips Produced by Inline Hot Rolling 

**Figure 3:**
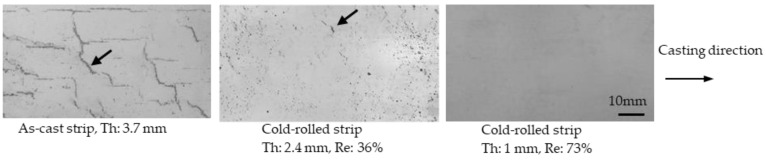
Surface condition of the as-cast strip and cold-rolled strips. Penetrant tests were conducted on the surfaces. Arrows show cracks. Th: strip thickness; Re: reduction.

Penetrant tests were conducted on the strips to check for cracking. Cracks existed on the surface on the cast strip, as shown in Figure 3. When the 3.7 mm thick cast strip was cold-rolled with a 36% thickness reduction to 2.4 mm, most of the large cracks disappeared, and only small cracks remained. When the strip was cold-rolled down to 1 mm, cracking was not observed. 

**Figure 4:**
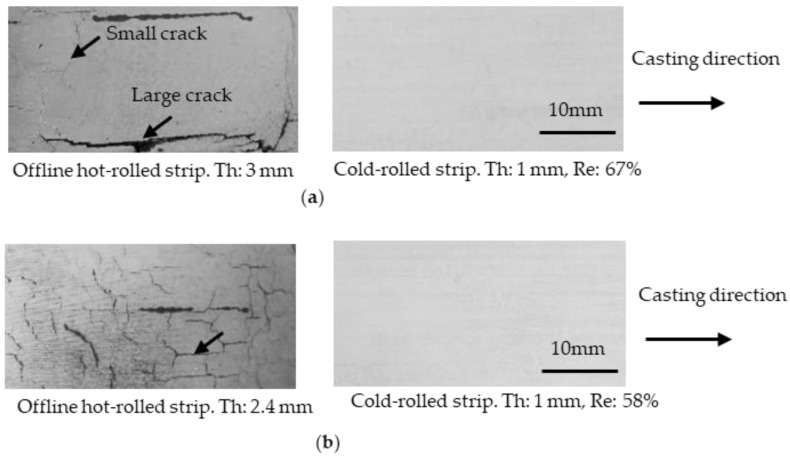
Surface condition of strips after offline hot rolling followed by cold rolling. (**a**) the reduction in offline hot rolling was 19 %. (**b**) the reduction in off line hot rolling was 36 %. The offline hot rolling temperature was 450 °C. Penetrant tests were conducted on the surfaces. Arrows show cracks. Th: strip thickness; Re: reduction.

The cast strips were subjected to OHR at 450 °C at thickness reductions of 19 and 36%, and were then cold-rolled down to 1 mm. The surfaces of the OHR + cold-rolled strips are shown in Figure 4. Large and small cracks were present on the surface of the OHR strip for the 19% reduction, and only small cracks remained when the reduction was 36%. The number of cracks decreased as the reduction increased. It is speculated that when cold rolling was conducted, the cracks were pressed and stretched, the depth of the cracks decreased, and finally, the cracks were closed. The effect of the thickness reduction on the reduction in surface cracking by OHR was inferior to that by cold rolling, based on the strip surfaces for the 36% thickness reduction shown in Figure 3 and Figure 4. The cause is not clear at this stage. For the OHR strips cold-rolled down to 1 mm, no cracks were observed. 

**Figure 5:**
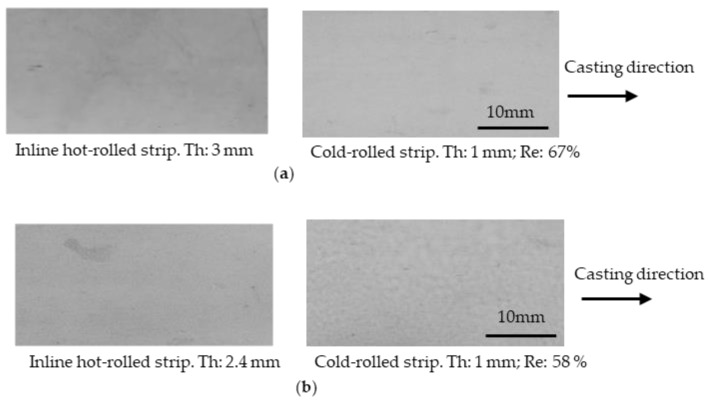
Surface condition of strips after inline hot rolling followed by cold rolling. (**a**) the reduction in inline hot rolling was 19 %. (**b**) the reduction in inline hot rolling was 36%. The inline hot rolling temperature was 450 °C. Penetrant tests were conducted on surfaces. Th: strip thickness. Re: reduction.

The surfaces of the IHR + the cold-rolled strips are shown in Figure 5. No cracks were observed for thickness reductions of 19 and 36%. IHR was remarkably effective in reducing surface cracking compared with cold rolling and OHR, although the cause is not clear. When IHR was conducted, the temperature on the inside of the strip was higher than that on the surface, suggesting that the inside of the strip might be softer than the surface. This might cause the reduction in cracking. The influence of surface crack oxidation on the reduction in cracking is not clear. In clad strip casting, when the cast strip is cooled to room temperature and heated, the oxide is hard, and bonding is not possible [33]. However, when the temperature falls but is still higher than 400 °C, the oxide is not hard, and bonding is possible [34]. 

In IHR, the surfaces of cracks might be more easily bonded to each other than in OHR. During OHR, oxidation on the crack surfaces may prevent the cracks from bonding. When the IHR strips were cold-rolled down to 1 mm, cracks did not appear. This means that IHR has a beneficial effect on the surface condition by removing surface cracks on a cast strip. The number of surface cracks could be reduced even by one-time IHR. A thickness reduction of 19% may be sufficient to reduce surface cracking.

### 3.3. Anisotropy of Strips Cold-Rolled from Cast Strip

**Figure 6:**
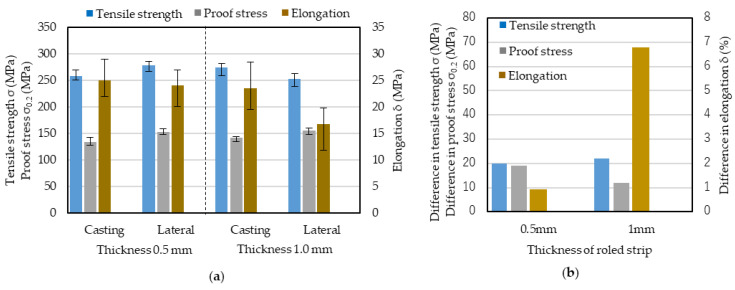
Results of tensile tests of cold-rolled 0.5 and 1.0 mm thicknesses. (**a**) results of tensile tests. (**b**) difference in mechanical properties between casting and lateral directions. Cast strips were cold-rolled down to 0.5 and 1.0 mm. Cold-rolled strips were annealed at 360 °C for 90 min, after which the tensile tests were conducted. Casting: casting direction; Lateral: lateral direction.

Cold-rolled cast aluminum strips are known to have anisotropic mechanical properties. The anisotropy of Al-5%Mg was checked before IHR was conducted. Tensile tests were conducted in the casting and lateral directions. Cast strips were cold-rolled down to 0.5 and 1 mm, corresponding to thickness reductions of 86.4 and 72.9%, respectively. These cold-rolled strips were annealed at 360 °C for 90 min, after which tensile tests were conducted. The results of the tensile tests are shown in Figure 6a for the casting and lateral directions for strips 0.5 and 1 mm thick, and the magnitude of the difference between the mechanical properties is shown in Figure 6b. Although differences exist for both strip thicknesses, the difference in elongation (0.8%) is much smaller for a thickness of 0.5 mm than that (6.8%) for a thickness of 1 mm. The effect of the thickness reduction in differences in the tensile strength and proof stress tests between the casting and lateral directions were not so large compared to that on the elongation. This may indicate that that when the thickness reduction is insufficiently large, the elongation is anisotropic. When a 3.7 mm thick cast strip was cold-rolled down to 0.5 mm, the thickness reduction was 86.4%. This means that a thickness reduction in more than about 85% may be needed to make the elongation isotropic. The thickness of a strip cast using a high-speed twin-roll caster is usually less than 4 mm. This means that to achieve an 85 % reduction in strip thickness, the final cold-rolled strip thickness should be about 0.6 mm. The strips used for structural materials used in cars and buildings are usually thicker than 1 mm. Thus, a process for reducing the anisotropy of roll-cast strips thicker than 1 mm is needed. In this study, the effectiveness of IHR for improving the anisotropy for a 1 mm thick IHR strip was investigated. 

### 3.4. Effect of the Thickness Reduction and Rolling Temperature during Inline Hot Rolling on Mechanical Properties

The effectiveness of IHR for aluminum alloys has not been well studied. It is considered that both the thickness reduction and temperature are important factors for IHR, and they affect the mechanical properties. To investigate this, tensile tests were conducted in the casting and lateral directions for the strips produced by IHR + cold rolling, with thickness reductions of 19, 27, and 36% and temperatures of 350 and 450 °C. Before the tensile tests, the strips were annealed at 350 °C for 90 min. 

**Figure 7:**
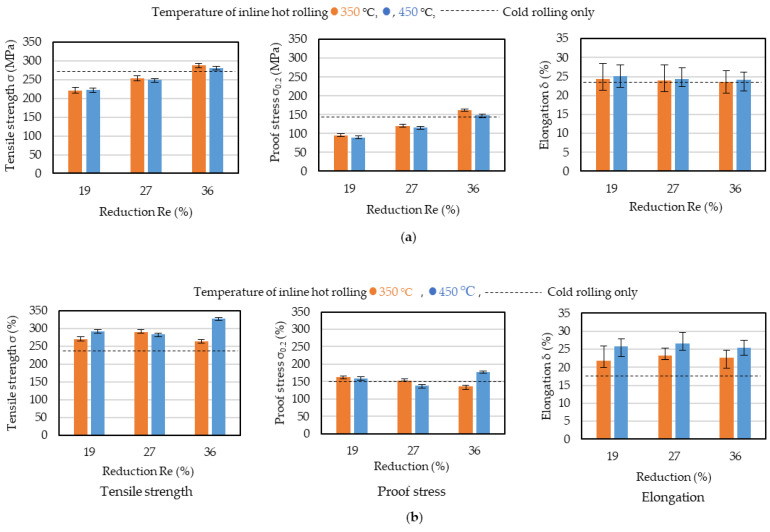
Results of tensile testing of inline hot-rolled strips. (**a**) Casting direction. (**b**) Lateral direction. Inline hot-rolled strips were cold-rolled down to 1 mm.

The results of these tests are shown in Figure 7. The horizontal dashed lines show the results for cold rolling only (CRO). The thickness reduction and the rolling temperature during IHR clearly influenced the mechanical properties, and the results depended on the testing direction. In the casting direction, the tensile stress and the proof stress increased as the thickness reduction increased, but there was no influence of the rolling temperature. The tensile stress and the proof stress for THR + cold rolling were similar to those for CRO when the IHR thickness reduction was 36%. In the lateral direction, the tensile strength following IHR + cold rolling was greater than that following CRO, regardless of the thickness reduction. The tensile strength for strips produced by IHR at 350 and 450 °C was greater than that for strips produced by CRO in the ranges of 15–30 MPa and 50–60 MPa, respectively. The proof stress for strips produced by IHR + cold rolling was almost the same as that for strips produced by CRO. In the casting direction, the elongation was not influenced by the thickness reduction or the temperature, and the elongation for strips produced by IHR + cold rolling was almost the same as that for strips produced by CRO. In the lateral direction, the elongation for strips produced by IHR + cold rolling was much greater than that for strips produced by the CRO. The elongation for strips cast at 450 °C was greater than that for those cast at 350 °C. The elongation in the lateral direction was much smaller than that in the casting direction for strips produced by CRO. The small elongation in the lateral direction could be improved by IHR. 

### 3.5. Effect of Inline Hot Rolling on the Anisotropy of Mechanical Properties 

**Figure 8:**
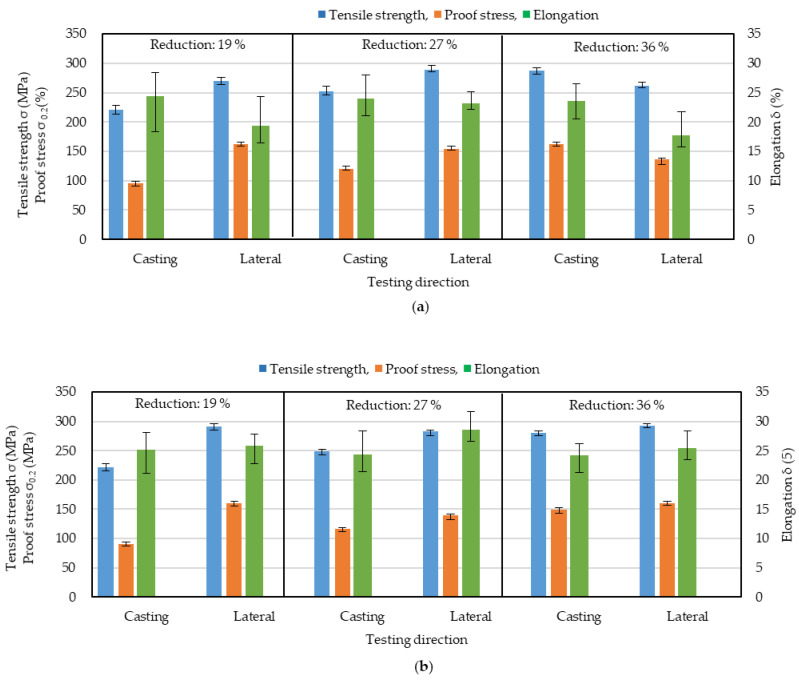
Effect of inline hot rolling reduction and temperature on results of tensile testing in the casting direction and the lateral direction. (**a**) inline hot rolling temperature was 350 °C. (**b**) Inline hot rolling temperature was 450 °C.

Figure 8 shows the results of tensile tests in the casting and lateral directions for the strips produced by IHR for different thickness reductions and temperatures. The mechanical properties in the casting and lateral directions were anisotropic at both temperatures. The lower elongation in the lateral direction was improved by IHR, as shown in Figure 7. This shows that IHR is effective in reducing anisotropy but not in reducing the anisotropy between the casting and lateral directions. 

**Figure 9:**
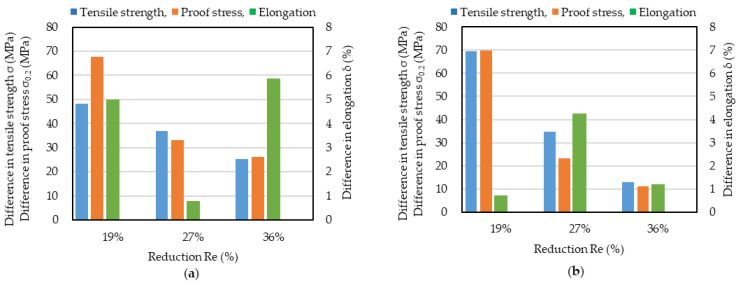
Difference in mechanical properties between the casting and lateral directions. (**a**) rolling temperature was 350 °C. (**b**) rolling temperature was 450 °C.

Figure 9 shows the differences in mechanical properties (absolute values) between the casting and lateral directions. The elongation difference was smaller for the following three conditions: rolling temperature of 350 °C and thickness reduction of 27%, rolling temperature of 450 °C and thickness reduction of 19%, and rolling temperature of 450 °C and thickness reduction of 36%. The differences in tensile stress and proof stress show that the appropriate conditions are a rolling temperature of 450 °C and a thickness reduction of 36%. Under these conditions, the tensile stress, proof stress, and elongation are larger than those for other conditions, including those for CRO. The differences in the tensile stress, proof stress, and elongation were 13 MPa, 11 MPa, and 1.2 %, respectively. Clearly, IHR is useful for reducing the anisotropy of the mechanical properties and also improving them. 

**Figure 10:**
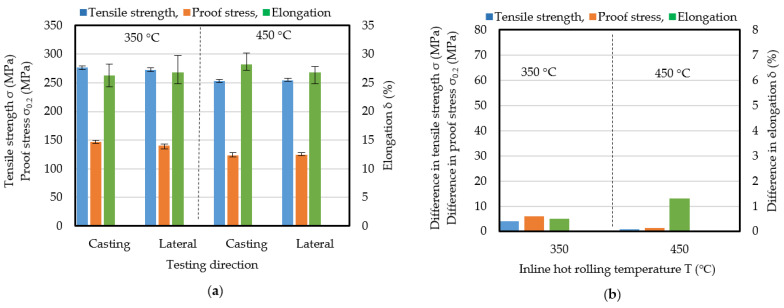
Results of tensile testing of the 0.5 mm thick strip made by inline hot rolling and cold rolling, and the difference in the mechanical properties between the casting and lateral directions. (**a**) result of tensile testing. (**b**) Difference in mechanical property between the casting and lateral direction. The rolling reduction was 27 %.

Because IHR was effective in reducing the anisotropy of 1 mm thick strips, its effect on 0.5 mm thick strips was investigated. The anisotropy of 0.5 mm thick strips produced by IHR + cold rolling may be reduced because the anisotropy for 0.5 mm thick strips was less than that for 1 mm thick strips produced by CRO, as shown in Figure 6. The results of tensile tests of 0.5 mm thick strips produced by IHR with a thickness reduction of 27% and temperatures of 350 and 450 °C in the casting and the lateral directions are shown in Figure 10. As seen in Figure 10a, the tensile strength and proof stress were smaller than those for strips produced by CRO, and the elongation was larger. Differences in the mechanical properties in the casting and lateral directions for strips produced by IHR + cold rolling were less than those for strips produced by CRO. The anisotropy was reduced by IHR, and the effect of IHR on the 0.5 mm thick strips was greater than that on the 1 mm thick strips. When the IHR temperature was 450 °C, the tensile stress and the proof stress decreased compared with those at 350 °C. The annealing time was long for strips produced by IHR at 450 °C, and grain growth might have occurred. 

### 3.6. Effect of Offline Hot Rolling on the Anisotropy of Mechanical Properties

**Figure 11:**
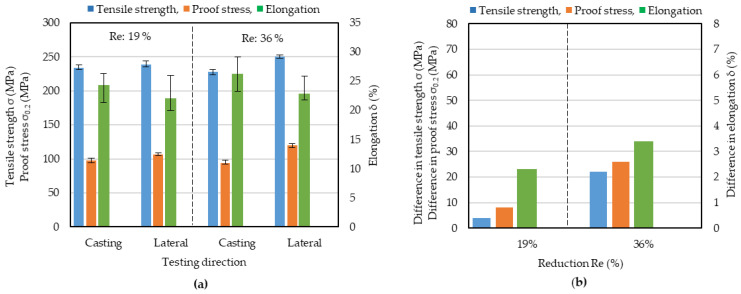
Results of tensile testing of offline hot-rolled and cold-rolled strips, and the difference in mechanical properties between the casting and lateral directions. (**a**) result of tensile testing. (**b**) difference in mechanical properties between the casting and lateral directions. The hot rolling temperature was 450 °C. Re: hot rolling reduction.

IHR has been shown to be effective in reducing the anisotropy of mechanical properties. Since this may also be the case for OHR, it is necessary to determine which hot rolling method is most effective. Strips produced by OHR at 450 °C and thickness reductions of 19 or 36% were cold-rolled down to 1 mm and annealed, and their mechanical properties were investigated in tensile tests. The results are shown in Figure 11 together with the differences between the casting and lateral directions. A comparison of Figure 6a and Figure 11a shows that the elongation in the lateral direction increased and the difference in elongation between the casting and lateral directions decreased in strips produced by OHR + cold rolling. The tensile strength and the proof stress are seen to be smaller than those for strips produced by CRO. The annealing time for the strip produced by OHR might have been inappropriately long. The differences in the mechanical properties between the casting and lateral directions for strips produced by OHR are shown in Figure 11b. When the thickness reduction was 36%, the anisotropy was greater than that when the thickness reduction was 19%. The tensile strength and proof stress in the casting direction decreased, and those in the lateral direction increased, when the thickness reduction was 36%; as a result, the anisotropy increased. The elongation increased in both directions, but the increase in the casting direction was larger than that in the lateral direction, so that the anisotropy increased.

For a rolling temperature of 450 °C and a thickness reduction of 36%, the tensile strength, the proof stress, and the elongation of the strips produced by OHR + cold rolling were compared with those for strips produced by IHR + cold rolling. A comparison of Figure 8b, Figure 9b and Figure 11 shows that the tensile strength, the proof stress, and the elongation for IHR + cold-rolled strips were greater, and their anisotropies were small. This shows that IHR is more effective than OHR at the reducing the anisotropy; moreover, the mechanical properties were effectively improved. 

It is considered that eliminating the hot rolling process is an advantage of twin-roll casting since it shortens the process. However, hot rolling, especially IHR, may be essential for reducing the isotropy of mechanical properties. 

### 3.7. Microstructure

**Figure 12:**
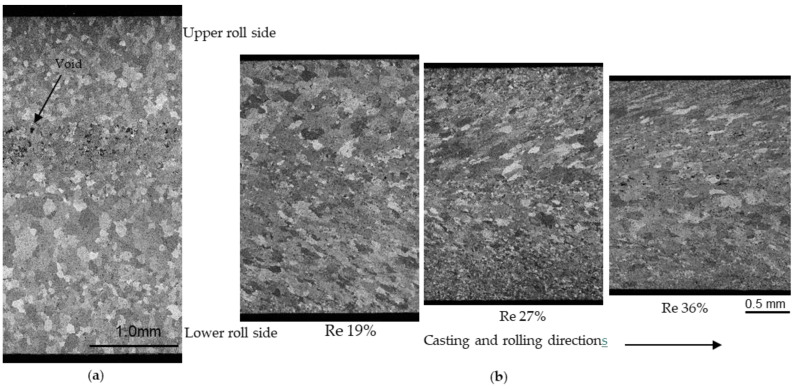
Cross-sections of the cast strip and inline hot-rolled strips. (**a**) as-cast strip. (**b**) inline hot-rolled strips. Rolling temperature: 450 °C; Re: reduction.

Cross-sectional optical micrographs of the as-cast and IHR strips are shown Figure 12. In the as-cast strip [Figure 12a], the layer solidified by the upper roll was thinner and the grain size was smaller than in the case of the lower roll. The difference in the solidified layer thickness was caused by the difference in solidification length between the upper and lower rolls. The molten metal temperature may have affected the grain size. It is considered that the temperature of the molten metal near the roll site was lower than that near the back dam plate. As a result, the grains in the layer solidified by the upper roll were smaller than those in the layer solidified by the lower roll. It is also likely that voids were produced by the melting of segregated Mg by the etching between the solidified layers. The number of such voids decreased as a result of the IHR process, as shown in Figure 12b, which may imply that segregation was improved. The grain morphology and void distribution of the as-cast and IHR strips are different. In addition, dynamic recovery and recrystallization also occur during IHR, which affects the anisotropy of mechanical properties. At this stage, it is not clear whether the reduction in mechanical anisotropy for the IHR strips is related to these underlying factors, and further research is needed. The IHR process reduced the grain size near the surface in the rolling direction. The grains in the strips produced by IHR were small and may have been formed by recrystallization. It is not clear when this recrystallization occurred, for example, during the IHR process or during cooling in air after IHR. The recrystallized grains near the surface were smaller, probably as a result of increased grain refinement with increasing thickness reduction. The shear strain was larger near the surface than in the interior, and it increased with increasing thickness reduction. The shear strain may have caused recrystallization to form finer grains. In this study, the strips were allowed to cool naturally to room temperature after IHR, but if forced cooling was applied, the grains may become even smaller. 

**Figure 13:**
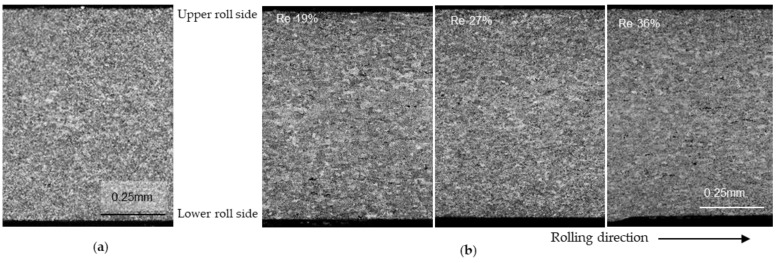
Cross-sections of strips cold-rolled down to 1 mm from the cast strip or inline hot-rolled strips. (**a**) cast strip. (**b**) inline hot-rolled strip. Cold-rolled strips were annealed at 360 °C for 90 min. Re: reduction in inline hot rolling.

Cross-sections of the strips produced by CRO and IHR + cold rolling are shown in Figure 13a,b, respectively. The strips were annealed at 360 °C for 90 min. The grain size of the cold-rolled and annealed strip was smaller than that for the as-cast or IHR strip. The grain size of the CRO strip seemed smaller than that for the IHR + the cold-rolled strips. No correlation was found between the grain size and the anisotropy of the mechanical properties. The thickness reduction in the IHR strips did not influence the microstructure of the cold-rolled or annealed strips. No clear correlation was found between the microstructure and the mechanical properties. Further research, for example, on texture, is needed to clarify the cause of the reduction in anisotropy caused by IHR.

## 4. Conclusions

IHR of Al-5%Mg strips cast using a UDTRC at a roll speed of 30 m/min was investigated. At a distance of 20 m between the caster and the hot rolling mill, the cast strip took 40 s to move from the caster to the hot rolling mill, and the temperature of the strip 40 s after casting was 483 °C. This temperature is high enough for hot rolling when the strip reaches the hot rolling mill. The 30 m/min casting speed is very fast, which contributed to attaining a strip temperature of 483 °C. This shows that IHR using a UCTRC is possible. The experimental equipment in this study modeled the IHR method.

The effectiveness of IHR in reducing surface cracking was investigated. Surface cracks on the cast strip could be erased by a 19% thickness reduction in the IHR method. This reduction was not sufficient to improve the surface cracks in the CRO or OHR strips.

The mechanical properties of the strip produced by CRO were not the same in the casting and lateral directions. In particular, the difference in elongation was remarkable, being much smaller in the lateral direction than in the casting direction. The effectiveness of IHR in reducing the elongation anisotropy was investigated. When the IHR temperature was 450 °C and the thickness reduction was 36%, the elongation in the casting and lateral directions was 24.2 and 25.4%, respectively, thus almost eliminating the anisotropy. The tensile strength in the casting and lateral directions was 280.2 and 293.3 MPa, respectively. The proof stress in the casting and lateral directions was 149.5 and 160.3 MPa, respectively. In the CRO strip, the elongation in the casting and lateral directions was 23.5 and 16.8%, respectively. The tensile strength in the casting and lateral directions was 274.2 and 252.4 MPa, respectively, and the proof stress was 141.1 and 155.0 MPa, respectively. The anisotropy of the tensile strength, the proof stress, and the elongation was lower for IHR, and the tensile strength, proof stress, and elongation were larger than those for CRO. In addition, the mechanical properties obtained by IHR were superior to those obtained by the OHR process. It became clear that IHR not only decreases the elongation anisotropy but also increases the tensile strength, proof stress, and elongation. 

## Figures and Tables

**Table 1:** Chemical composition of Al-5%Mg (mass%).

Cu	Si	Mg	Zn	Fe	Mn	Ni	Ti	Cr	Al
0.02	0.10	4.86	0.01	0.16	0.44	0.00	0.00	0.03	Bal.

## Data Availability

Data are contained within the article.
